# Retraction: Atorvastatin Improves Survival in Septic Rats: Effect on Tissue Inflammatory Pathway and on Insulin Signaling

**DOI:** 10.1371/journal.pone.0159283

**Published:** 2016-07-08

**Authors:** Kelly Lima Calisto, Bruno de Melo Carvalho, Eduardo Rochete Ropelle, Francine Cappa Mittestainer, Angélica Costa Aranha Camacho, Dioze Guadagnini, José Barreto Campelo Carvalheira, Mario José Abdalla Saad

The authors and editors retract this publication [1] following an investigation into concerns around the data presented in several figures that were brought to the editors’ attention.

After the publication of the article, concerns were raised about Figures 2,4, 5 and 6. The authors stated that the wrong blots were included in several of the published figures and that the p-JNK blots in Figure 4B were inadvertently duplicated from a figure in an earlier publication. A Correction [2] was issued to address the errors identified. Following the publication of the Correction, additional concerns were raised regarding figures included in the original article and in the Correction. Upon follow up with the authors, the editors remain concerned about the following panels in Figure 2:

- Figure 2D - B-actin: This panel duplicates the B-actin panel in Figure 2A.
- Figure 2F- B-actin: The first band from the original blot for this figure was not included in the published panel.
- Figure 2I - B-actin: Upon evaluation of the raw blot supplied by the author, the editors are concerned that the evidence supplied does not support the results reported in this figure.

An institutional inquiry has been undertaken at the University of Campinas (UNICAMP) in São Paulo, Brazil and while the investigation acknowledged errors in the preparation of the figures, they did not find evidence of research misconduct. However, the preparation of the figures falls below the standard of publication and therefore the authors and the editors have agreed that the correct action is to retract the article. The authors apologize to the scientific community and will seek to publish a corrected manuscript version corroborating the findings of this work.
